# Determinants of Inhaler Choice at Hospital Discharge

**DOI:** 10.3390/medsci14010081

**Published:** 2026-02-11

**Authors:** Myriam Calle Rubio, Soha Esmaili, Iman Esmaili, Pedro José Adami Teppa, Miriam García Carro, José Carlos Tallón Martínez, Consolación Riesco Rubio, Laura Fernández Cortés, María Morales Dueñas, Valeria Chamorro del Barrio, Juan Luis Rodríguez Hermosa, Jorge García Aragón

**Affiliations:** 1 Department of Medicine, School of Medicine, Universidad Complutense de Madrid, 28040 Madrid, Spain; mcallerubio@gmail.com (M.C.R.); soha@esmaili.ws (S.E.);; 2 Pulmonology Department, Hospital Clínico San Carlos, 28040 Madrid, Spainlfcortes@salud.madrid.org (L.F.C.); valeria.chamorro@salud.madrid.org (V.C.d.B.); 3 Instituto de Investigación Sanitaria del Hospital Clínico San Carlos (IdISSC), 28040 Madrid, Spain; 4 CIBER de Enfermedades Respiratorias (CIBERES), 28040 Madrid, Spain; 5 Pulmonology Department, Hospital Universitario La Zarzuela, Hospital Quirónsalud San Jose, 28023 Madrid, Spain; 6 School of Medicine, Universidad Antonio de Nebrija, 28248 Madrid, Spain; 7 ISNS Data Analytics and Research, Vancouver, BC V6Z 1Y6, Canada; 8 Pulmonology Department, Hospital Central de La Defensa Gómez Ulla, 28047 Madrid, Spain; adamipedro92@gmail.com; 9 Farmacy Department, Hospital Clínico San Carlos, 28040 Madrid, Spain

**Keywords:** COPD, Asthma, inhaler device selection, clinical inertia, peak inspiratory flow, hospital readmission

## Abstract

**Background**: Inhaler device changes at hospital discharge should address patient capacity yet often reflect routine. We evaluated the appropriateness of these decisions and their impact on clinical outcomes. **Methods**: In this prospective observational study (*N* = 480), we assessed patient technical capacity using a composite of critical errors, inspiratory flow, adherence, and knowledge. We stratified patients into ‘Need-Positive’ and ‘Need-Negative’ cohorts to quantify patterns of clinical inertia and over-adjustment. Multivariable models identified predictors of decision-making and associations with 30-day outcomes. **Results**: Device changes were primarily determined by the pre-admission device class (spacers: aOR 0.52; 95% CI 0.28–0.96; *p* = 0.037) and by the patient’s treatment pathway rather than by clinical need. This disconnect generated two types of errors: 38.3% of Need-Positive patients (*n* = 214) experienced clinical inertia (no corrective action), while 36.8% of Need-Negative patients (*n* = 266) underwent over-adjustment (unnecessary switching). Inertia perpetuated errors in patients with need, whereas over-adjustment was associated with the emergence of new errors in patients without need. Successful mismatch resolution was associated with a significantly lower 30-day readmission rate (12.1% vs. 32.5%; OR 0.48; 95% CI 0.26–0.88; *p* = 0.017). **Conclusions**: Discharge prescribing is driven more by habit than by objective assessment, leading to widespread missed opportunities for correction. Implementing evidence-based protocols to identify and resolve patient–device mismatches may represent a high-impact strategy to reduce readmissions and associated healthcare use.

## 1. Introduction

Inhaled therapies are the cornerstone of management for chronic airway diseases, conditions that impose a substantial global burden of morbidity and mortality [1]. While the direct delivery of medication to the lungs aims to maximize efficacy, a persistent gap exists between the potential of these treatments and their real-world effectiveness [2,3]. This disparity is a primary driver of poor clinical control, fueling a preventable cycle of exacerbations and increased healthcare costs [4]. A key mechanism underlying this therapeutic failure is the high prevalence of critical technique errors, which can severely compromise drug delivery [5].

Although patient-level “need indicators”—such as physiological mismatches, poor adherence, or deficient knowledge—are established barriers to effective treatment [6,7], the point of hospital discharge represents a pivotal, yet under-investigated, opportunity to correct them. Decisions made at this juncture are critical, but it remains unclear whether they are driven by objective patient need or by pre-existing “treatment pathway” factors, such as institutional routines or the passive renewal of prior devices [8].

This uncertainty masks two fundamental failures in clinical practice: clinical inertia, where necessary changes are not made despite clear evidence of need, and over-adjustment, where devices are switched without a documented indication [9]. Understanding the true drivers of these decisions is essential for developing interventions that can bridge the gap between evidence and practice [10]. Therefore, this study aimed to determine whether discharge prescribing is guided by objective patient capacity or by prior treatment routines. Specifically, we sought to quantify patterns of inertia and over-adjustment, examine the moderating influence of medical specialty and patient education, and—crucially—evaluate whether resolving technical mismatches is associated with improved clinical outcomes, including the reduction in hospital readmissions.

## 2. Materials and Methods

### 2.1. Study Design and Population

This study was a retrospective analysis of a prospectively collected, observational cohort from the AIRE (Real-World Evidence on the Use of Inhalers) project [11]. The study was conducted between March 2023 and March 2024 at a large, tertiary university hospital in Madrid, Spain, and adheres to the STROBE guidelines for observational studies [12]. The source population comprised all adult patients (≥18 years) admitted to medical inpatient services who were prescribed inhaled therapy for chronic airway diseases, specifically COPD and asthma. Patients were included regardless of the primary reason for admission (respiratory or non-respiratory), provided they had a documented history of established chronic inhaler use (≥3 months) prior to admission and complete discharge prescription data. Exclusion criteria were severe cognitive impairment precluding reliable assessment, death during hospitalization, or discharge to palliative care.

### 2.2. Measurements and Definitions

Data were obtained from two primary sources: the institutional electronic health record (EHR) and a structured, in-person assessment. Variables extracted from the EHR included patient demographics, comorbidities (quantified using the Charlson Comorbidity Index [13]), admitting service (Pulmonology vs. Internal Medicine/Other), and detailed pharmacological history (pre-admission and in-hospital device classes). The in-person assessment was conducted by trained respiratory nurses who were blinded to the subsequent clinical decisions to prevent observation bias. This assessment evaluated four key indicators of technical adequacy: critical inhaler errors (identified using device-specific checklists); Peak Inspiratory Flow (PIF) mismatches (defined as PIF ≤ 30 L/min measured against resistance using In-Check DIAL in patients prescribed a dry powder inhaler); poor adherence (TAI score ≤ 45) [14]; and poor device knowledge (patient-reported inability). Prior inhaler training was assessed by patient recall and categorized as “Good” (formal instruction with demonstration), “Fair” (verbal instruction only), “Poor” (brief instruction), or “None” (self-taught).

Based on these indicators, a composite variable of ‘Any Mismatch’ was defined a priori as the presence of at least one of the above deficits. For appropriateness analyses, the population was stratified into a ‘Need-Positive’ cohort (patients with at least one documented mismatch) and a ‘Need-Negative’ cohort (patients with no mismatches). ‘Clinical Inertia’ was defined as the failure to change the device in a Need-Positive patient, whereas ‘Over-adjustment’ was defined as a device change in a Need-Negative patient. Finally, ‘Mismatch Resolution’ was defined as the technical correction of the specific documented issue by the new discharge prescription.

### 2.3. Statistical Analysis

Continuous variables were reported as medians [interquartile range] and compared using the Mann–Whitney U test, as they did not follow a normal distribution (verified via Shapiro–Wilk test). Categorical variables were compared using the χ^2^ test. To identify independent predictors of device change, multivariable logistic regression models were constructed. Variables with clinical relevance were entered into the core model, and we explicitly tested for interactions between documented need and potential moderators, specifically medical specialty and prior patient training. Model fit was assessed using the Akaike Information Criterion (AIC). Missing data were present in <5% of cases; therefore, a complete-case analysis approach was utilized.

To analyze the appropriateness of action, separate analyses were performed within the stratified ‘Need-Positive’ and ‘Need-Negative’ cohorts. The impact of clinical action on downstream outcomes (regimen simplification, mismatch resolution, and technique errors) was quantified using Absolute Risk Differences (RD) with 95% confidence intervals calculated via the Newcombe–Wilson hybrid score method. Finally, the prognostic impact of mismatch resolution on 30-day readmissions was assessed using logistic regression adjusted for age, comorbidity, and service. Analyses were performed using R software (version 4.2.2), with statistical significance defined as a two-sided *p*-value < 0.05.

### 2.4. Ethical Considerations

The study protocol was conducted in accordance with the Declaration of Helsinki and was approved by the Institutional Review Board of the Hospital Clínico San Carlos (CI:23/069-O_M_NoSP). Written informed consent was obtained from all participants prior to the in-person assessment and data collection. All patient data were de-identified prior to analysis to ensure confidentiality.

## 3. Results

### 3.1. Influence of Pathway Context on Inhaler Device Changes

Table 1 presents the baseline demographic, clinical, and contextual characteristics of the study cohort (*N* = 480), stratified by whether patients underwent an inhaler device change at hospital discharge. The two groups were comparable in terms of demographic factors, including age (*p* = 0.471) and Charlson comorbidity burden (*p* = 0.586).

However, significant differences were observed in treatment pathway factors. The complexity of the pre-admission regimen was associated with device changes; notably, patients using Soft Mist Inhalers (SMI) prior to admission were exclusively found in the device change group (30.3% vs. 0.0%), whereas those maintained on Dry Powder Inhalers (DPI) were more frequently in the no-change group (46.6% vs. 34.1%; *p* < 0.001). Similarly, the number of devices used at home differed significantly, with patients using ≥2 devices being more prevalent in the change group (*p* < 0.001). In-hospital device management also varied, with Nebulization being equally common, but spacer use being less frequent in the change group.

Regarding patient-reported measures, while adherence scores (TAI) were similar (*p* = 0.911), patients who experienced a device change reported significantly higher baseline inhaler tolerance (“Good” tolerance: 96.2% vs. 83.5%; *p* = 0.005) but poorer training quality recall (*p* = 0.034).

### 3.2. Factors Associated with Device Change at Discharge

Multivariable logistic regression models were analyzed to identify independent factors associated with device switching. In the fully adjusted model (Table 2), pathway factors demonstrated the strongest statistical associations. Patients admitted on dry powder inhalers (aOR 0.49; 95% CI 0.31–0.77; *p* = 0.002) or spacers (aOR 0.52; 95% CI 0.28–0.96; *p* = 0.037) had significantly lower odds of undergoing a device change compared to those using pMDIs. Advanced age was also independently associated with a lower likelihood of switching (aOR 0.92 per 10-year increase; 95% CI 0.86–0.99; *p* = 0.025).

**Figure 1:**
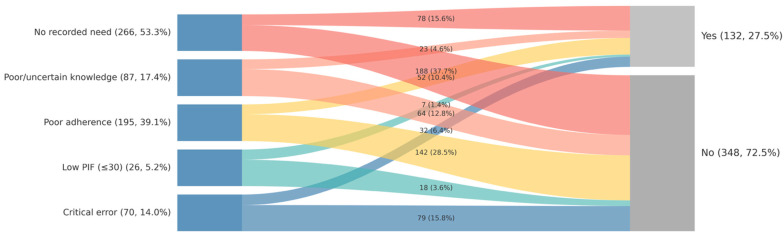
Patient Flow from Prioritized Need Indicators to Clinical Action at Discharge. Note. The diagram illustrates the flow of patients from their primary documented need indicator (left) to the clinical action taken at discharge (right). Need indicators were prioritized hierarchically to assign each patient to a single mutually exclusive category. The width of the flows is proportional to the number of patients (*N* = 480). aOR = adjusted odds ratio; CI = confidence interval; pMDI = pressurized metered-dose inhaler; PIF = peak inspiratory flow.

In contrast, patient-level need indicators did not drive clinical decisions. As visually demonstrated in Figure 1, neither critical inhaler errors nor low inspiratory flow showed a clear directional association with device changes. Their inclusion in the regression models did not significantly improve model fit (*p* = 0.261), confirming a disconnect between identified clinical needs and the therapeutic action taken at discharge.

### 3.3. Influence of Clinical Specialty and Prior Training on Decision-Making

Beyond the main predictors, interaction analyses examined whether decision-making varied by clinical setting or patient history. The clinical specialty appeared to modify responsiveness: probability of switching was approximately 10 percentage points higher in Pulmonology services compared to Internal Medicine when a mismatch was present (*p* = 0.057).

**Figure 2:**
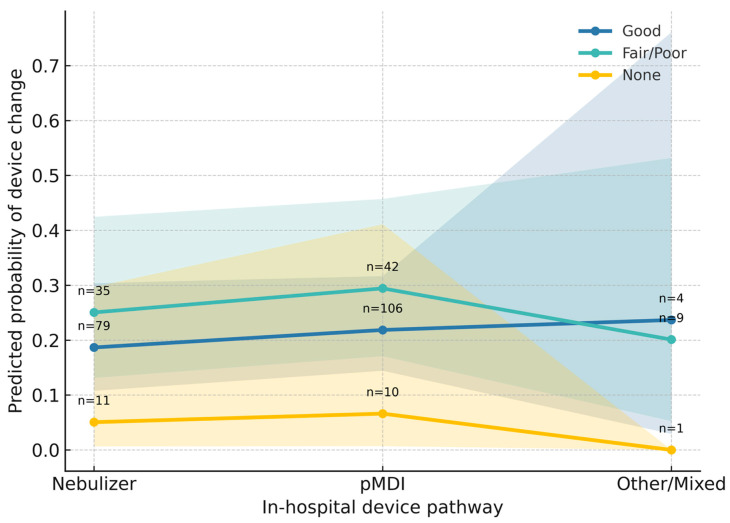
Interaction between Patient Training and Treatment Pathway on Device Change. Note. 95. confidence intervals. The consistent flatness of the ‘None’ trajectory (yellow) illustrates that lack of prior training acts as a barrier to device switching, whereas prior training facilitates change. (Data source: Analysis based on the *N* = 435 patients with available education data.).

Crucially, the quality of prior patient training significantly moderated the decision to change devices (*p* < 0.001 for interaction). As illustrated in Figure 2, patients who had never received prior training (Yellow line) exhibited a consistently low probability of switching, suggesting a pattern of inertia where clinicians hesitate to modify therapy in training-naïve patients. In contrast, those with prior training showed higher switching rates, a gap that widened notably in complex pathway scenarios.

### 3.4. Appropriateness of Clinical Action: Inertia Versus Over-Adjustment

**Figure 3:**
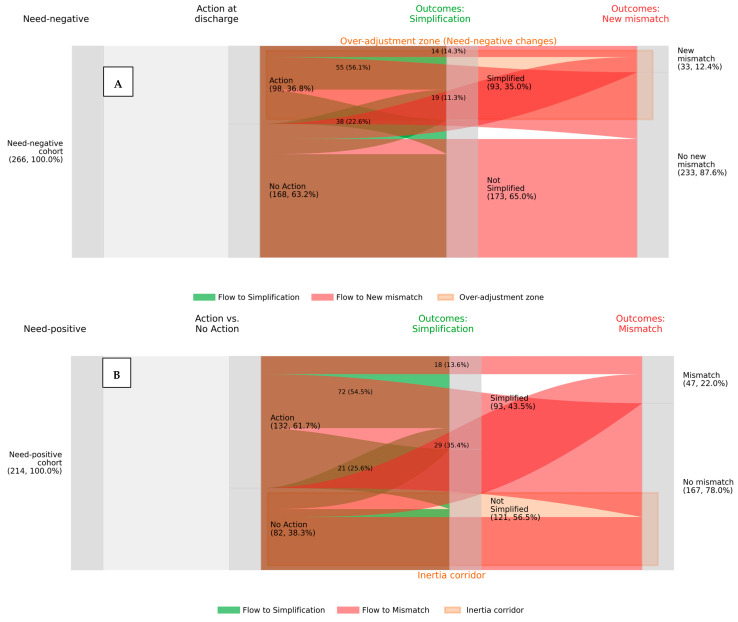
Divergent Consequences of Clinical Action. Note. The Sankey diagrams track patient trajectories from cohort entry (left) through clinical action (middle) to discharge outcomes (right). (**A**) Need-Negative Cohort (Over-Adjustment): Illustrates that clinical action in patients without a documented need (top flows) frequently results in new technique mismatches (red flows) with minimal gain in simplification. The orange shaded zone highlights the “Over-adjustment zone”. (**B**) Need-Positive Cohort (Clinical Inertia): Illustrates the consequence of inaction. The bottom beige flow (“Inertia corridor”) shows patients with needs who received no action, leading to persistent mismatches. In contrast, appropriate action (top flows) is strongly linked to regimen simplification (green flows). Green flows = Simplification; Red flows = Flow to Mismatch.

To evaluate the clinical appropriateness of these decisions, we stratified the cohort by their need status. The divergence between clinical action and documented need is visualized in Figure 3, which contrasts the downstream trajectories of patients with and without a confirmed indication for change.

Clinical Inertia (Need-Positive Cohort).

Among patients with a documented need (*n* = 214), 38.3% received no corrective action (Figure 3B, bottom flow). We conducted a multivariable analysis to identify drivers of this inertia. While no single factor reached strict statistical significance, the quality of patient training showed the strongest signal: patients who recalled “Good” prior training showed a protective trend against inertia compared to those with poor or no training (aOR 0.49; 95% CI 0.21–1.14; *p* = 0.096), suggesting that clinicians may be more empowered to act on patients who are already educated.

Over-Adjustment (Need-Negative Cohort).

Conversely, among patients without documented technical need for the device (*n* = 266), 36.8% underwent a device change (Figure 3A, top flow). As visually tracked in Figure 3A, this unprompted switching was associated with new technique mismatches in 19.4% of patients (red flows), compared to 0.0% in patients who remained on their baseline therapy. Multivariable analysis identified two significant independent predictors of this unprompted switching: patients under the care of the Pulmonology service (aOR 1.88; 95% CI 1.02–3.46; *p* = 0.043) and those using a DPI prior to admission (aOR 2.01; 95% CI 1.08–3.72; *p* = 0.028) were significantly more likely to experience a device change without a documented technical indication.

Quantifying the Clinical Impact.

The downstream clinical consequences of these decisions are quantified in Table 3. In the ‘Need-Positive’ cohort, the decision to intervene was strongly justified by outcomes: patients who received the indicated device change achieved a 43.9% absolute risk increase in regimen simplification and a 73.2% resolution rate of mismatches compared to those who experienced inertia.

In stark contrast, for the ‘Need-Negative’ cohort, the decision to change the device conferred no measurable benefit. The rate of regimen simplification was identical between those who changed and those who did not (Risk Difference +1.1%; 95% CI −10.6 to +12.8). Furthermore, the unprompted change was associated with potential harms, including a 19.4% absolute increase in new technique errors and a significant reduction in good adherence at follow-up (Risk Difference −14.6%).

### 3.5. Clinical Impact: Mismatch Resolution and Readmissions

**Figure 4:**
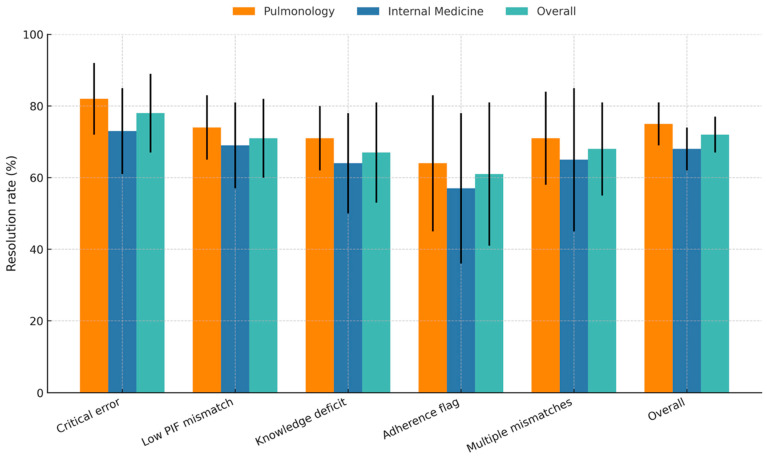
Mismatch Resolution Rates by Type and Medical Specialty. Note. The bar chart shows the percentage of documented mismatches that were successfully resolved following a device change. Error bars represent 95% confidence intervals. The ‘Overall’ category represents the aggregated resolution rate (≈73.5%) across all mismatch types for the cohort of mismatch-positive patients who received a device change (*N* = 132). PIF = Peak Inspiratory Flow.

Finally, we assessed the effectiveness of device changes when they were applied to resolve specific problems. Among patients with identified mismatches, the overall resolution rate was 73.5% (95% CI 65–78%), although effectiveness varied by clinical setting. As shown in Figure 4, Pulmonology services achieved consistently higher point estimates for resolution across all mismatch types compared to Internal Medicine, suggesting a pattern of greater technical proficiency in the specialized setting.

Prognostic Value of Mismatch Resolution.

Beyond technical correction, successfully resolving a mismatch was associated with tangible clinical benefits at the 30-day follow-up (Table 4). Patients whose mismatches were corrected experienced a statistically significant reduction in 30-day readmissions (12.1% vs. 32.5%; OR 0.48, 95% CI 0.26–0.88; *p* = 0.017). Furthermore, successful resolution was significantly associated with higher rates of good adherence compared to those with unresolved issues (78.8% vs. 61.5%; *p* = 0.004). Patients whose mismatches were successfully resolved had higher adherence rates and lower healthcare utilization at 30 days compared with those whose mismatches persisted.

## 4. Discussion

This study deconstructs the decision-making process surrounding inhaler device changes at hospital discharge, revealing a critical disconnect between clinical action and documented needs in patients’ inhaler use. Our central finding is that these decisions were overwhelmingly driven by the existing treatment pathway—specifically the device class used prior to admission and patient age—rather than by actionable indicators of mismatch such as critical technique errors or insufficient inspiratory flow. This dominance of established treatment pathways over evidence resulted in two paradoxical patterns: a widespread failure to act when a change was warranted (Clinical Inertia), and conversely, a frequent tendency to change devices without a documented reason related to device use (Over-Adjustment). Crucially, we show that this disconnect was associated with a significant reduction in 30-day readmissions when mismatches were resolved, supporting device alignment as a potentially high-impact safety strategy.

### 4.1. The Primacy of Context over Need

Our analysis indicates that the strongest predictors of a device change were non-clinical factors. Patients admitted on a DPI or spacer were significantly less likely to be switched compared to pMDI users, independent of their technical proficiency or physiological capacity. This finding aligns with literature on therapeutic inertia, where established prescribing patterns often outweigh individualized assessment [15]. While prior studies have highlighted the prevalence of inhaler misuse [16,17], our work quantifies the “invisibility” of these errors in the decision-making matrix. As visually demonstrated in Figure 1, indicators of need failed to trigger a consistent clinical response, suggesting that discharge decisions follow the path of regimen continuity rather than an evidence-based reassessment. Studies of chronic disease management at discharge show that medication changes are often not sustained, and that outpatient providers may reverse inpatient decisions, reflecting a lack of coordinated, patient-centered reassessment [18]; however, our analysis was anchored to the discharge decision point to specifically evaluate the appropriateness of the hospital intervention, distinct from subsequent outpatient modifications.

Qualitative studies also describe that time pressures during hospital discharge can constrain individualized assessment, potentially reducing opportunities to optimize therapy [19]. Furthermore, the literature highlights that lack of access to inhalers, such as fixed dual bronchodilator therapy, in hospital formularies can lead to inappropriate use of inhaled corticosteroids [20]. The literature consistently emphasizes the need for multidisciplinary collaboration and improved communication to overcome inertia and ensure discharge decisions are truly evidence-based and tailored to individual patient needs. This is consistent with the broader literature emphasising the importance of individualised selection of inhalers, taking into account patient factors such as cognitive function, manual dexterity, inspiratory flow, and knowledge of the device, as well as the need for ongoing education on inhalation technique.

### 4.2. The Duality of Misalignment: Inertia and Over-Adjustment

Our analysis identifies widespread clinical inertia. In nearly 40% of patients with a documented error, no corrective action was taken, showing that physicians were significantly less likely to change the device in patients with no history of prior training. This may reflect greater reluctance to introduce a new device when training has been limited, which can inadvertently perpetuate the use of an inappropriate inhaler. While education at discharge is the primary strategy for knowledge deficits, it cannot resolve physiological constraints (e.g., low peak inspiratory flow) or physical inability, which mandate a device change. The guidelines consistently recommend patient-centred device selection and periodic technique verification to ensure optimal drug delivery and clinical outcomes [21,22]. However, the time and resources required for comprehensive device training are often limited in real-world practice, especially during transitions of care or in hospital settings with high staff turnover. In addition, the wide variety of inhaler devices available poses significant challenges for prescribers in selecting and teaching the optimal device. System-level barriers, such as hospital formulary restrictions, can further limit device choice and reinforce reliance on familiar and readily available devices, even if they are not perfectly suited to the patient’s needs [23,24,25]. All these factors can contribute to therapeutic inertia, the tendency to maintain existing therapy rather than re-evaluate and optimise it, even after discharge. Failure to follow these recommendations, whether due to physician reluctance or system-level barriers, can result in the continued use of an inappropriate device, undermining both adherence and therapeutic efficacy.

Secondly, we observed a pattern of excessive adjustment. In the “Negative Need” cohort, more than one-third of stable patients underwent a device change. Our analysis identified pulmonary care and pre-admission use of DPI as predictors of this unsolicited change. This may reflect a pharmacological strategy, such as switching to triple therapy combinations available only on specific devices, rather than a technical necessity. However, our analysis shows that these changes were associated with a net increase in new technical errors, indicating that routine changes should not be made in patients who do not show signs of device misuse. The real-life impact of changing inhalers for non-clinical reasons (e.g., for economic or form-related reasons) can worsen disease control if adequate education and consent are not provided, increasing technical errors and reducing adherence [26,27]. Evidence supports that instruction and periodic review of technique are essential with each therapeutic change.

### 4.3. Clinical Impact and Prognostic Implications

The most pivotal finding of our study is the link between technical resolution and downstream outcomes. When mismatches were identified and resolved, patients experienced tangible benefits. As detailed in Table 4, successful resolution was associated with a 52% reduction in the odds of 30-day readmission (OR 0.48) and significantly higher adherence. This positions inhaler selection as a patient-safety-relevant decision point rather than a matter of “preference.” It suggests that failing to address a mismatch may represent a missed opportunity with measurable clinical consequences. Recent real-world studies and expert reviews further demonstrate that inappropriate device selection and poor technique are common, leading to suboptimal disease management, increased exacerbations, and reduced quality of life [23,28,29]. In the line with this, systematic screening and intervention at discharge may represent a high-value strategy associated with reduced acute care utilization [23,30,31,32]. While specialist services showed numerically higher resolution rates (Figure 4), the overlap in confidence intervals suggests that systematic protocols are needed across all medical services [27,33]. Structured in-hospital interventions have been shown to reduce the proportion of misused inhalers at discharge by over 40%, with corresponding reductions in readmission rates and acute care utilization [34,35].

### 4.4. Limitations

Our study has limitations. First, its observational design precludes definitive causal inference; unmeasured confounders, such as formulary restrictions or physician preference, may have influenced decisions. Second, appropriateness was defined based on recorded data; the absence of a documented “need” does not definitively rule out unrecorded issues, although we used a comprehensive composite assessment to minimize misclassification. Third, while interaction analyses generated hypothesis-generating insights regarding training and specialty, these should be validated in larger cohorts. In addition, our outcome analysis was limited to all-cause readmissions, and we did not objectively re-verify inhaler technique post-discharge. Finally, as a single-center study, generalizability to systems with different discharge protocols should be confirmed.

### 4.5. Clinical Implications

The association between mismatch resolution and reduced 30-day readmissions suggests that inhaler selection is a modifiable factor in patient safety. These findings support a shift in discharge protocols: from passive renewal of prior therapy to an active screening strategy that evaluates technique and inspiratory flow in all admitted patients. Further research is needed to determine whether an active screening strategy evaluating inhaler technique and inspiratory flow at discharge improves clinical outcomes, including exacerbation rates and readmissions, and to assess the impact of pharmacist-led interventions, such as electronic reminders and smart inhaler technology, in both hospital and outpatient settings [36,37]. Furthermore, the prevalence of ‘Over-Adjustment’ highlights the need for de-implementation strategies to curb unnecessary device switching in stable patients, reserving changes for cases with a documented technical indication or a pharmacological necessity. While our data demonstrate a reduction in acute care utilization, future pragmatic trials are needed to confirm whether bundled interventions—combining screening, decision support, and education—can sustain these benefits over the long term. Given the single-center design, multicenter validation is also warranted to determine whether the observed patterns of therapeutic inertia and over-adjustment are consistent across different healthcare systems.

## 5. Conclusions

Inhaler device changes at hospital discharge are currently driven by the treatment pathway and routine rather than by objective patient capacity. This disconnects results in dual patterns of inefficiency: clinical inertia, where patients with critical errors remain on unsuitable devices—often driven by a lack of prior training—and over-adjustment, where patients without documented need undergo switching. Crucially, we show that correcting these mismatches is not merely a procedural formality but may represent a high-yield strategy associated with a significant reduction in 30-day hospital readmissions. These findings highlight the urgent need to replace habit-based prescribing with evidence-based, patient-centered discharge protocols to improve clinical outcomes in respiratory care.

## Figures and Tables

**Table 1:** Baseline Characteristics of the Cohort by Device Change at Discharge (*N* = 480).

Characteristic	No Change (*n* = 348)	Change (*n* = 132)	Total (*N* = 480)	*p*-Value
Demographics				
Age (years), median [IQR]	78.5 [69.0–85.0]	77.0 [65.8–85.2]	78.0 [69.0–85.0]	0.471
Charlson comorbidity index, median [IQR]	3.0 [1.0–4.0]	3.0 [1.0–4.0]	3.0 [1.0–4.0]	0.586
Male sex, *n* (%)	173 (49.7)	61 (46.2)	234 (48.8)	0.833
System-level factors				
Service, *n* (%)				0.248
- Internal Medicine	188 (54.0)	62 (47.0)	250 (52.1)	
- Pulmonology	81 (23.3)	40 (30.3)	121 (25.2)	
- Other	79 (22.7)	30 (22.7)	109 (22.7)	
Pathway context				
Pre-admission device class, *n* (%)				<0.001
- DPI	162 (46.6)	45 (34.1)	207 (43.1)	
- pMDI	113 (32.5)	26 (19.7)	139 (29.0)	
- Spacer	73 (21.0)	21 (15.9)	94 (19.6)	
- SMI	0 (0.0)	40 (30.3)	40 (8.3)	
Pre-admission therapy (grouped), *n* (%)				<0.001
- LAMA	26 (7.5)	16 (12.1)	42 (8.8)	
- LABA	2 (0.6)	1 (0.8)	3 (0.6)	
- LAMA+LABA (fixed)	65 (18.7)	35 (26.5)	100 (20.8)	
- LABA+ICS (separate)	1 (0.3)	0 (0.0)	1 (0.2)	
- LABA+ICS (fixed)	92 (26.4)	22 (16.7)	114 (23.8)	
- Triple (separate)	35 (10.1)	28 (21.2)	63 (13.1)	
- Triple (fixed)	126 (36.2)	29 (22.0)	155 (32.3)	
- Other	1 (0.3)	1 (0.8)	2 (0.4)	
In-hospital device (grouped), *n* (%)				0.001
- Nebulization	147 (42.2)	56 (42.4)	203 (42.3)	
- MDI ± spacer	183 (52.6)	69 (52.3)	252 (52.5)	
- SMI	4 (1.1)	7 (5.3)	11 (2.3)	
- Other/Mixed	14 (4.0)	0 (0.0)	14 (2.9)	
Home device count, median [IQR]	1.0 [1.0–1.0]	1.0 [1.0–1.0]	1.0 [1.0–1.0]	0.001
- 1 device	306 (87.9)	100 (75.8)	406 (84.6)	
- ≥2 devices	42 (12.1)	32 (24.2)	74 (15.4)	
Months of inhaled treatment, median [IQR]	17 [7.0–46.0]	16 [8.0–38.0]	17 [7.0–45.0]	0.421
Need indicators				
Critical inhaler error present, *n* (%)	62 (17.8)	23 (17.4)	85 (17.7)	0.912
PIF (L/min), median [IQR]	60.0 [50.0–70.0]	55.0 [41.0–67.8]	60.0 [45.0–70.0]	0.148
PIF ≤ 30 vs. >30, *n* (%)				---
- ≤30 L/min	18 (8.0)	7 (8.1)	25 (8.0)	
- >30 L/min	208 (92.0)	79 (91.9)	287 (92.0)	
Low PIF–DPI mismatch, *n* (%)	34 (9.8)	11 (8.3)	45 (9.4)	0.711
Any mismatch composite, n (%)	91 (26.1)	31 (23.5)	122 (25.4)	0.553
Education and adherence context				
Patient training rating, *n* (%)				0.034
- Did not receive	20 (9.2)	2 (2.5)	22 (7.4)	
- Good	139 (63.8)	50 (63.3)	189 (63.6)	
- Fair	46 (21.1)	17 (21.5)	63 (21.2)	
- Poor	13 (6.0)	8 (10.1)	21 (7.1)	
- Very poor	0 (0.0)	2 (2.5)	2 (0.7)	
Adherence (TAI categories), *n* (%)				0.591
- Good	62 (30.4)	18 (25.7)	80 (29.2)	
- Intermediate	63 (30.9)	26 (37.1)	89 (32.5)	
- Poor	79 (38.7)	26 (37.1)	105 (38.3)	
TAI score, median [IQR]	48.0 [43.0–50.0]	48.0 [44.0–49.8]	48.0 [43.3–50.0]	0.911
Inhaler tolerance, *n* (%)				0.005
- Fair	32 (14.7)	1 (1.3)	33 (11.1)	
- Good	182 (83.5)	76 (96.2)	258 (86.9)	
- Poor	4 (1.8)	2 (2.5)	6 (2.0)	
Knowledge—indication, *n* (%)				0.934
- Good	175 (80.3)	60 (75.9)	235 (79.1)	

Note. Values are presented as *n* (%) for categorical variables and as median [interquartile range] for continuous variables. Group comparisons were performed using χ^2^ tests for categorical variables and Mann–Whitney U tests for continuous variables. Percentages are calculated using the number of patients with available data for each variable as the denominator. Device classes were defined according to pre-admission therapy records. DPI = dry powder inhaler; ICS = inhaled corticosteroid; LABA = long-acting beta2-agonist; LAMA = long-acting muscarinic antagonist; PIF = peak inspiratory flow. Low PIF–DPI mismatch was defined as PIF ≤ 30 L/min among patients prescribed a DPI. pMDI = pressurized metered-dose inhaler; SMI = soft mist inhaler. Adherence categories were based on the Test of Adherence to Inhalers (TAI), with “Good” (≥50), “Intermediate” (46–49), and “Poor” (≤45). Respiratory disease categories reflect pre-existing diagnoses recorded prior to the index admission.

**Table 2:** Adjusted Odds Ratios for Predictors of Device Change at Discharge (Main Multivariable Model; *N* = 480).

Predictor	aOR	95% CI	*p*-Value
Service (ref. Internal Medicine)			
Pulmonology	1.61	0.94–2.74	0.081
Other	1.15	0.66–1.98	0.622
Respiratory disease label (ref. None)			
COPD	0.57	0.29–1.13	0.110
Asthma	0.55	0.25–1.24	0.150
Bronchiectasis	0.92	0.37–2.31	0.863
Other/Unknown	1.02	0.41–2.55	0.965
Pre-admission device (ref. pMDI/SMI)			
Dry Powder Inhaler (DPI)	0.49	0.31–0.77	0.002
Spacer	0.52	0.28–0.96	0.037
In-hospital device (ref. Nebulization)			
MDI ± spacer	1.16	0.75–1.79	0.504
Soft Mist Inhaler (SMI)	4.74	1.39–16.18	0.013
Other/Mixed	0.00 *	—	<0.001
Age (per 10-year increase)	0.92	0.86–0.99	0.025

Note. The model was adjusted for age (per 10 years), Charlson comorbidity index, admitting service, respiratory disease class, pre-admission device class, and in-hospital device class. aOR = adjusted odds ratio; CI = confidence interval; COPD = chronic obstructive pulmonary disease; MDI = metered-dose inhaler; pMDI = pressurized metered-dose inhaler; SMI = soft mist inhaler. * The “Other/Mixed” in-hospital device group (*n* = 14, all no-change) produced complete separation, preventing the calculation of a standard odds ratio.

**Table 3:** Clinical Consequences of Device Changes Stratified by Need Status.

Outcome at Discharge	Action (*n* = 132)	No Action (*n* = 82)	Risk Diff (95% CI)
(A) NEED-POSITIVE COHORT			
(Comparison: Correction vs. Inertia)			
Regimen simplified to 1 device, *n* (%)	79 (59.8)	13 (15.9)	+43.9 (+30.3 to +57.5)
Mismatch resolved, n (%)	97 (73.5)	0 (0.0) *	+73.2 (+65.9 to +80.5)
Optimal technique achieved, *n* (%)	81 (61.4)	33 (40.2)	+21.2 (+7.8 to +34.6)
			
(B) NEED-NEGATIVE COHORT	(*n* = 98)	(*n* = 168)	
(Comparison: Over-Adjustment vs. Standard Care)			
Regimen simplified to 1 device, *n* (%)	18 (18.4)	29 (17.3)	+1.1 (−10.6 to +12.8)
New mismatch created, *n* (%)	19 (19.4)	0 (0.0) **	+19.4 (+11.5 to +27.3)
Adherence at follow-up (Good), *n* (%)	12 (12.2)	45 (26.8)	−14.6 (−26.6 to −2.6)

Note. The table contrasts the impact of clinical action in patients with a documented need (Panel A) versus those without (Panel B). Risk Diff = Risk Difference (Action rate minus No Action rate) calculated with 95% Newcombe confidence intervals. * In the Need-Positive No Action group, the documented mismatch persists by definition (0% resolution). ** In the Need-Negative No Action group, patients remained on their baseline adequate therapy, resulting in no new technique mismatches generated by hospital intervention.

**Table 4:** Downstream Clinical Outcomes by Mismatch Resolution Status (*N* = 242).

Outcome (30-Day Follow-Up)	Resolved (*n* = 165)	Not Resolved (*n* = 77)	OR [95% CI]	*p*-Value
30-day readmission, *n* (%)	20 (12.1%)	25 (32.5%)	0.48 [0.26–0.88]	0.017
Adherence at follow-up (Good), *n* (%)	130 (78.8%)	47 (61.5%)	2.36 [1.31–4.24]	0.004
Symptom improvement (CAT ≥ 2), *n* (%)	68 (41.2%)	26 (33.8%)	1.37 [0.81–2.34]	0.236

Note. Outcomes were measured at 30 days post-discharge. ORs = adjusted odds ratios, derived from logistic regression models adjusted for age, Charlson comorbidity index, and admitting service. Mismatch resolution status was determined at hospital discharge.

## Data Availability

The original contributions presented in the study are included in the article; the data presented in this study are available on request from the corresponding author.

## Abbreviations

The following abbreviations are used in this manuscript:

DPI	Dry powder inhaler
ICS	Inhaled corticosteroid
LABA	Long-acting beta2-agonist
LAMA	Long-acting muscarinic antagonist
PIF	Peak inspiratory flow
pMDI	Pressurized metered-dose inhaler
SMI	Soft mist inhaler
TAI	Test of Adherence to Inhalers
COPD	Chronic obstructive pulmonary disease
EHR	Institutional electronic health record
